# Association between plain-water consumption and suicide attempts among U.S. adolescents: Evidence from the 2017–2021 Youth Risk Behavior Survey

**DOI:** 10.1371/journal.pone.0359065

**Published:** 2026-10-05

**Authors:** Catalina Canizares, Francisco Cardozo, Abiraahmi Shankar, Raymond Balise, Pamela Morris-Perez

**Affiliations:** 1 Department of Applied Psychology, Steinhardt School of Culture, Education, and Human Development, New York University, New York, United States of America; 2 Department of Psychology, School of Social Sciences, Universiad de los Andes, Bogotá, Colombia; 3 Department of Psychiatry and Behavioral Sciences, Miller School of Medicine, University of Miami, Florida, United States of America; 4 Department of Public Health, Miller School of Medicine, University of Miami, Florida, United States of America; University of Huelva: Universidad de Huelva, SPAIN

## Abstract

**Background:**

Adolescent suicide attempts remain difficult to predict, in part because research has concentrated on a narrow set of risk factors. We examined whether self-reported plain-water consumption is associated with past-year suicide attempt among U.S. adolescents, whether that association is graded across levels of intake, and whether a zero-intake response adds information beyond established risk factors.

**Methods:**

We analyzed the 2017, 2019 and 2021 cycles of the U.S. national Youth Risk Behavior Survey (n = 36,779). Missing data, including the plain-water item, were multiply imputed (40 datasets) and pooled by Rubin’s rules, accounting for the survey design. The association was estimated unadjusted, then adjusted for demographics, each of six risk domains, and all domains together. Least absolute shrinkage and selection operator (LASSO) models, tuned under two metrics and two penalty rules, provided variable selection and held-out performance; odds ratios come from unpenalized models.

**Results:**

Reporting no plain water was uncommon (3.6%) and associated with past-year suicide attempt (survey-weighted odds ratio 2.14, 95% CI 1.70–2.71), in each cycle. The association was confined to the zero category: the adjusted odds ratio for none was 2.55 (1.91–3.39) against 3 times/day, whereas among those reporting any plain water there was no gradient (1.01, 0.97–1.06). It remained associated after adjustment for each domain separately (1.45–2.01) but attenuated to 1.34 (0.97–1.85) under simultaneous adjustment. LASSO retained it in every model at the discrimination optimum and, under the one-standard-error rule, in five of six domains but not the full model. Adding it changed held-out discrimination by at most 0.0011.

**Conclusions:**

Reporting no plain-water consumption in the past seven days was consistently associated with higher odds of a past-year suicide attempt among U.S. adolescents. However, the association was confined to the zero-water category, attenuated after simultaneous adjustment for all risk domains, and did not improve prediction. These findings suggest that the zero-water response may function as a behavioral marker of broader risk rather than as an independent risk factor.

## Introduction

Adolescent suicide attempts are a critical public health concern in the United States. Data from the Youth Risk Behavior Surveillance System (YRBSS) reveal that approximately 9% of adolescents attempted suicide in the past year. This statistic is alarming, given the well-established link between suicide attempts and death by suicide, underscoring the urgent need to better understand the factors associated with these behaviors. Research on risk factors for death by suicide and suicide attempts dates back to 1965 and has increased exponentially throughout the years [1]. However, despite the amount of research, the ability to accurately predict suicide attempts is limited, barely better than chance, and it is still a challenge [1–3].

A major limitation in the study of suicide attempt risk factors is the ongoing focus on a narrow set of predictors across both empirical and theoretical studies, often replicated across various populations. Empirical research consistently links suicide attempts with prior suicide attempts, schizophrenia, depression, borderline personality disorder, previous psychiatric hospitalizations, and nonsuicidal self-injury [1]. Popular theoretical models echo this emphasis, with explanations predominantly centered on individual-level factors. For example, the Interpersonal Theory of Suicide (IPTS) [4], the Three-Step Theory of Suicide [5], and the Integrated Motivational-Volitional Model [6] all highlight internal processes like cognition and emotion as key in understanding suicide attempts. According to IPTS, individuals are at the greatest risk when they simultaneously experience thwarted belongingness and perceived burdensomeness [4]. The Three-Step Theory of Suicide posits that pain, hopelessness, connectedness, and capacity for suicide together contribute to the emergence of both the desire for suicide and eventual suicide attempts [5]. Similarly, the Integrated Motivational-Volitional Model suggests that defeat, entrapment, acquired capability, and access to lethal means interact to create a high-risk environment for suicide [6].

More recently, researchers have begun to examine broader socio-environmental and ecological influences on suicide. For instance, studies have investigated the impact of urbanicity, with some reporting significant associations between suicide and factors such as proximity to urban centers, subway access, and residential density [7,8], while others have found no significant relationship [9]. Although relatively few studies on environmental factors of suicide have focused on youth populations, a systematic review by Ching and colleagues [10] identified air pollution, drought, and rising temperatures as key environmental stressors linked to increased suicide risk among adolescents.

In addition to these physical environmental factors, recent work has highlighted how everyday behaviors such as dietary patterns may shape suicide risk. Studies have found that frequent fast-food consumption, low intake of fruits and vegetables, and higher consumption of caffeinated and sugary beverages are associated with greater likelihood of suicidal ideation, planning, and attempts among adolescents, even after adjusting for known confounders such as age, sex, race, and depressive symptoms [11–13].

Despite growing interest in how diet and environmental exposures shape mental health, one everyday behavior has received comparatively little attention in adolescent suicide research, plain-water consumption. From a developmental science perspective, environmental conditions and everyday exposures are integral to the ecological systems in which youth develop and should be integrated into models of wellbeing [14]. Evidence at the individual level makes plain-water consumption a plausible candidate for investigation. Experimentally induced mild dehydration degrades mood and increases fatigue, tension, and difficulty concentrating in healthy young adults [15,16]. In a large adult sample, drinking more plain water was associated with lower odds of depression and anxiety [17]. Among 112,250 Korean adolescents, drinking less than one glass per day was associated with higher odds of depression, suicidal ideation, suicide planning and suicide attempt [18]. Low plain-water consumption also travels with other markers of adversity: among US high school students it clusters with poor academic grades, physical inactivity, and poor dietary behaviors [19], and tap water avoidance is closely tied to household food insecurity and to racial and economic disadvantage [20].

We accordingly treat not drinking plain water as a behavioral indicator rather than a physiological exposure, one that may mark disengagement from routine self-care, symptom burden, or constrained material circumstances, without presuming which of these predominates. A more distant possibility, raised here only as a hypothesis for future work, is that water carries some biological signal, ecological studies link naturally occurring lithium in community water supplies to lower suicide rates in adults [21,22]. Those studies measure lithium concentrations in water systems rather than individual drinking behavior, and they cannot speak to the behavior examined here.

What has not been examined is how plain-water consumption behaves when it is placed in direct competition with the risk factors that dominate adolescent suicide research. Prior individual-level studies adjust for a modest set of covariates, which leaves open whether the association is distinct from, or simply absorbed by, the established predictors of adolescent suicide attempts.

The present study addresses this gap by testing whether self-reported plain-water consumption is retained as a predictor of suicide attempts when placed in direct competition with established risk factors across multiple domains. Using data from the 2017, 2019 and 2021 cycles of the YRBSS, we fit seven domain-specific Least Absolute Shrinkage and Selection Operator (LASSO) regression models, each organized around a theoretically defined category of suicide risk: substance use, trauma and victimization, dietary habits, physical activity and sleep, suicidal ideation and distress, risk behaviors, and a full model combining all domains. In each model, plain-water consumption is included alongside predictors from that domain, even when it does not conceptually belong to the domain. This cross-domain design functions as a stress test: if plain-water consumption is retained by LASSO even within domains where it is conceptually extraneous, this would be consistent with the indicator carrying information not already captured by those risk factors. Retention under penalization is not on its own evidence of independent or clinical importance, since it depends on the penalty rule, on correlations among predictors, and on coding choices, and we therefore treat it as one piece of evidence rather than as the answer. Alongside this stress test we ask two further questions that bear on how any such signal should be interpreted. The first concerns the shape of the association: whether risk falls steadily as reported consumption rises, or is instead confined to adolescents who report drinking no plain water at all. The second concerns its usefulness: whether the indicator improves out-of-sample prediction of suicide attempts beyond established risk factors. The study is exploratory and cross-sectional throughout, and we frame it as hypothesis-generating.

## Materials and methods

### Design and data source

We conducted a cross-sectional analysis of the 2017, 2019 and 2021 national Youth Risk Behavior Survey (YRBS), because these are the most recent consecutive cycles that administered every measure this analysis uses. The YRBS is a biennial, school-based survey of students in grades 9–12 attending public and private schools in the United States, using a three-stage cluster sample designed to produce nationally representative estimates. Survey methodology is described in detail elsewhere [23]. The data are publicly available and de-identified, so the analysis did not constitute human-subjects research requiring review. Respondents missing the suicide-attempt item were excluded, leaving 36,779 in the analytic sample. Missingness on predictors, including the plain-water item, was handled by imputation, so every analysis below uses the same respondents.

### Measures

#### Suicide attempt.

The outcome was derived from the item asking how many times the respondent actually attempted suicide during the past 12 months, dichotomized as any attempt versus none.

#### Plain-water consumption.

The YRBS asks, “During the past 7 days, how many times did you drink a bottle or glass of plain water? (Count tap, bottled, and unflavored sparkling water.)” Seven ordered response options run from “I did not drink water during the past 7 days” through “4 or more times per day.” We analyzed this item in two forms. The **binary indicator**, contrasting “I did not drink water during the past 7 days” against any reported consumption, is the primary exposure and is the form used unless stated otherwise. The **ordinal item** retains the original seven levels and serves to test whether risk is graded across the consumption distribution.

#### Domain predictors.

Forty-seven predictors were organized into six theoretically defined domains. Substance use (11 items): current cigarette, cigar, marijuana, alcohol, vaping and smokeless tobacco use; lifetime heroin, synthetic marijuana, cocaine and methamphetamine use; and being offered drugs at school. Trauma and victimization (9 items): feeling unsafe at school, being threatened at school, physical fighting at school, forced sexual intercourse, sexual violence, sexual abuse by a partner, physical dating violence, bullying, and cyberbullying. Dietary habits (10 items): no intake of fruit juice, fruit, salad, potatoes, carrots, other vegetables, soda, milk, breakfast or sports drinks. Physical activity and sleep (7 items): physical activity, three or more hours of television, three or more hours of video games, physical education attendance, sports team participation, sports-related concussion and eight or more hours of sleep. Suicidal ideation and distress (4 items): suicidal ideation, suicide planning, hopelessness and difficulty concentrating. Risk behaviors (6 items): seatbelt use, riding with a drinking driver, driving after drinking, texting while driving, weapon carrying at school and gun carrying.

#### Demographic covariates.

All adjusted models included sex, race/ethnicity, grade, sexual orientation, Hispanic/Latino ethnicity and survey year.

#### Harmonization across cycles.

Because question numbering changes between cycles, each variable was located by its description in the cycle-specific codebook rather than by item number. Derived indicators whose definition changed between cycles were rebuilt from the underlying ordinal item, so that every variable carries a single definition throughout.

### Missing data

Missingness on the plain-water item was 19.7% and rose across cycles, from 12.0% in 2017 to 13.6% in 2019 and 29.1% in 2021. It differed by outcome: 25.6% among adolescents reporting an attempt against 19.0% among those not. The 8,895 respondents excluded for a missing outcome (19.5%) were enriched for the exposure: 11.1% reported no plain water against 3.5% of the analytic sample. Differential missingness was not confined to the plain-water item (S1 and S2 Tables in S1 File).

Multiple imputation by chained equations was performed with the mice package [24], generating 40 datasets with 20 iterations. The predictor matrix was specified rather than selected by a correlation threshold: every imputation model contained the outcome, the plain-water item, the demographic covariates, survey year and the sampling weight, and was completed with the twenty variables most strongly associated with either the target variable or with its missingness, giving 28–29 predictors per imputed variable. The primary sampling unit and stratum identifiers were held out of the imputation model and reattached afterward for the design-based analyses.

The plain-water item was imputed in its seven-level ordinal form by proportional-odds regression; the remaining categorical variables were imputed by polytomous regression and binary variables by logistic regression. All subsequent models were fitted on these imputed datasets. Convergence diagnostics are shown in S1 Fig in S1 File and a comparison of the imputed and observed distributions of the ordinal item in S2 Fig and S3 Table in S1 File.

Because missingness on the plain-water item was not independent of the outcome, we did not rest on the missing-at-random assumption alone. A sensitivity analysis retaining a missing plain-water response as its own category, rather than imputing it, is reported in S4 Table in S1 File.

### Analytic strategy

We used two complementary modeling approaches: unpenalized logistic regression to estimate the association, and LASSO logistic regression to evaluate whether the water indicator survived competition with established risk factors and whether it improved prediction. All odds ratios reported below come from unpenalized models, because LASSO shrinks coefficients toward zero and confidence intervals from penalized fits lack a standard interpretation. Unpenalized models were fitted once per imputed dataset and pooled by Rubin’s rules with Barnard-Rubin degrees of freedom, and accounted for the YRBS design through sampling weights, primary sampling units and strata.

#### Association.

We estimated the association between the binary no-water indicator and past-year suicide attempt, first unadjusted and then adjusted for the demographic covariates. To test whether risk was graded across the consumption distribution rather than concentrated at zero, we repeated the adjusted model with the seven-level ordinal item in place of the binary indicator, taking 3 times/day as the reference category because it had the lowest observed attempt prevalence. We then tested for a gradient above zero by estimating the trend per additional time per day among adolescents who reported drinking any plain water; because the exposure was imputed, the membership of that subgroup varied across imputations. The adjusted binary model was also fitted separately within each cycle. Unweighted estimates are reported alongside the design-based ones for these models, and in the supplement for the domain-adjusted models described below.

#### Adjustment against the risk domains.

The demographically adjusted model described above served as the baseline. We then added each risk domain to it separately, and finally all six simultaneously. Every model in the series was fitted on the same imputed datasets, and therefore on the same respondents, so the change in the odds ratio from one model to the next reflects the covariates that were added and not a loss of respondents to missing data. Interactions between the water indicator and physical activity and sports team participation, and estimates within strata of both, are reported in the supplement.

#### Penalized models and prediction.

The sample was split before imputation, stratified on the outcome and holding out 25% of respondents, and the two partitions were imputed separately so that no information from the held-out set could enter the training imputation model. LASSO models (L1 penalty) were fitted for each of seven specifications, the six risk domains and all domains combined, twice per specification with and without the water indicator, once on each of the 40 imputed training datasets. The contrast of interest is whether the water indicator is retained under each of two penalty-selection rules. The discrimination-optimal rule takes the penalty with the best cross-validated performance. The one-standard-error rule takes the largest penalty whose performance remains within one standard error of that optimum, and therefore keeps fewer predictors. Cross-validation was used only to choose the penalty within each imputation. Agreement across the 40 imputations is reported as a check that the pattern is not an artifact of a single imputed draw [25]. These models were fitted without survey weights because they serve to compare predictive performance rather than to estimate population parameters. Categorical predictors were dummy coded and zero-variance columns removed. The penalty was tuned by 5-fold cross-validation stratified on the outcome, over a grid of 50 log-spaced values from 10^−8^ to 1, under two tuning metrics (ROC-AUC and average precision). For every fit we recorded whether the selected penalty fell at a grid boundary. Models were trained at the natural outcome prevalence without resampling [26].

Each fitted model was applied to the matching imputation of the held-out partition. We report discrimination (ROC-AUC, pooled on the logit scale, and average precision), the Brier score, and calibration intercept and slope. Calibration was assessed by logistic recalibration: on the held-out data we regressed the outcome on the linear predictor from the training fit. A slope of 1 means predicted risks are neither too extreme nor too modest; a slope below 1 means they are too spread out [27,28]. Sensitivity, specificity, predictive values and the proportion of students flagged at five operating points are reported in the supplement. Incremental value was assessed by comparing the paired models with and without the water indicator within each imputation using DeLong’s test and pooling the difference.

Analyses were conducted in R 4.6.1 using tidymodels, glmnet, mice, survey, pROC and broom.

## Results

### Sample

Across the three cycles, 36,779 respondents had the suicide-attempt item observed, of whom 3,657 reported an attempt (survey-weighted prevalence 8.9%). The plain-water item was observed for 29,543 of these (80.3%). Reporting no plain water in the past seven days was uncommon: 1,047 respondents, 3.1% of those with the item observed after weighting. The sample was split into 27,584 training and 9,195 held-out respondents, with unweighted attempt prevalences of 10.1% and 9.5% respectively. Table 1 shows the sample composition.

**Table 1:** Sample characteristics by past-year suicide attempt status, pooled 2017-2021 national YRBS (N = 36,779).

	No attempt (n = 33,122)		Attempt (n = 3,657)	
Characteristic	Unweighted n (%)^1^	Weighted %^2^	Unweighted n (%)^1^	Weighted %^2^
**Year**				
2017	9,849 (29.7%)	32.3%	837 (22.9%)	26.3%
2019	9,453 (28.5%)	28.9%	1,067 (29.2%)	28.9%
2021	13,820 (41.7%)	38.8%	1,753 (47.9%)	44.8%
**Sex**				
Female	16,088 (49.0%)	48.6%	2,295 (64.6%)	64.8%
Male	16,733 (51.0%)	51.4%	1,255 (35.4%)	35.2%
Missing	301	0.8%	107	3.0%
**Race**				
Am Indian/Alaska Native	254 (0.8%)	0.5%	56 (1.6%)	1.0%
Asian	1,642 (5.1%)	4.7%	126 (3.6%)	3.4%
Black or African American	4,157 (12.8%)	10.1%	646 (18.3%)	14.6%
Hispanic/Latino	2,721 (8.4%)	9.1%	239 (6.8%)	8.0%
Multiple-Hispanic	4,468 (13.8%)	14.8%	589 (16.7%)	17.5%
Multiple-Non-Hispanic	1,780 (5.5%)	5.3%	271 (7.7%)	7.2%
Native Hawaiian/Other PI	188 (0.6%)	0.5%	24 (0.7%)	0.6%
White	17,191 (53.1%)	55.0%	1,575 (44.7%)	47.6%
Missing	721	2.1%	131	3.0%
**Grade**				
9	8,635 (26.3%)	26.4%	1,073 (29.8%)	30.0%
10	8,571 (26.1%)	25.4%	1,030 (28.7%)	27.7%
11	8,151 (24.8%)	24.5%	807 (22.4%)	21.8%
12	7,518 (22.9%)	23.8%	685 (19.1%)	20.5%
Missing	247	0.7%	62	1.8%
**Sexual orientation**				
Bisexual	2,621 (8.5%)	8.5%	865 (25.9%)	28.8%
Gay or Lesbian	795 (2.6%)	2.5%	216 (6.5%)	6.1%
Heterosexual	26,346 (85.2%)	85.3%	1,962 (58.8%)	56.5%
Not sure	1,163 (3.8%)	3.7%	294 (8.8%)	8.7%
Missing	2,197	7.8%	320	8.3%
**Plain water, past 7 days**				
None	856 (3.2%)	2.8%	191 (7.0%)	6.3%
1-3 times/week	2,284 (8.5%)	8.0%	287 (10.5%)	9.6%
4-6 times/week	2,931 (10.9%)	10.8%	326 (12.0%)	11.3%
1 time/day	2,157 (8.0%)	8.0%	204 (7.5%)	7.1%
2 times/day	4,123 (15.4%)	15.4%	355 (13.0%)	12.9%
3 times/day	4,701 (17.5%)	17.8%	365 (13.4%)	13.6%
4+ times/day	9,769 (36.4%)	37.3%	994 (36.5%)	39.3%
Missing	6,301	15%	935	19%

^1^n (%)

^2^%

Unweighted n (%) is the respondent count and the column percentage in the analytic sample. Weighted % is the survey-weighted column percentage. Weighted population counts are omitted because they estimate the U.S. high-school population rather than the sample.

#### The zero-water response is associated with suicide attempt.

Averaged across imputations, 17.7% of the 1,337 adolescents reporting no plain water in the past seven days reported a past-year suicide attempt, compared with 9.7% of the 35,442 reporting any. The survey-weighted odds ratio is 2.14 (95% CI 1.70–2.71, p < 0.001), and adjustment for sex, race/ethnicity, grade, sexual orientation, Hispanic/Latino ethnicity and survey year leaves it at 2.10 (1.65–2.67). Unweighted estimates are smaller and more precise but of the same direction (unadjusted 2.01, 1.72–2.36; adjusted 1.90, 1.61–2.24).

The association was present in every cycle taken separately, with survey-weighted adjusted odds ratios of 2.77 (1.86–4.14) in 2017, 2.17 (1.47–3.21) in 2019 and 1.69 (1.10–2.59) in 2021 (unweighted 2.39, 1.98 and 1.58). The point estimates decline monotonically, while missingness on the plain-water item rises from 12.0% in 2017 to 29.1% in 2021.

### The association is confined to the zero category

Modeling the full seven-level item showed that the association did not extend across the consumption distribution (Fig 1). Taking 3 times/day as the reference, the survey-weighted adjusted odds ratio for reporting no plain water is 2.55 (1.91–3.39), while the remaining six categories are clustered between 1.10 and 1.34.

**Fig 1:**
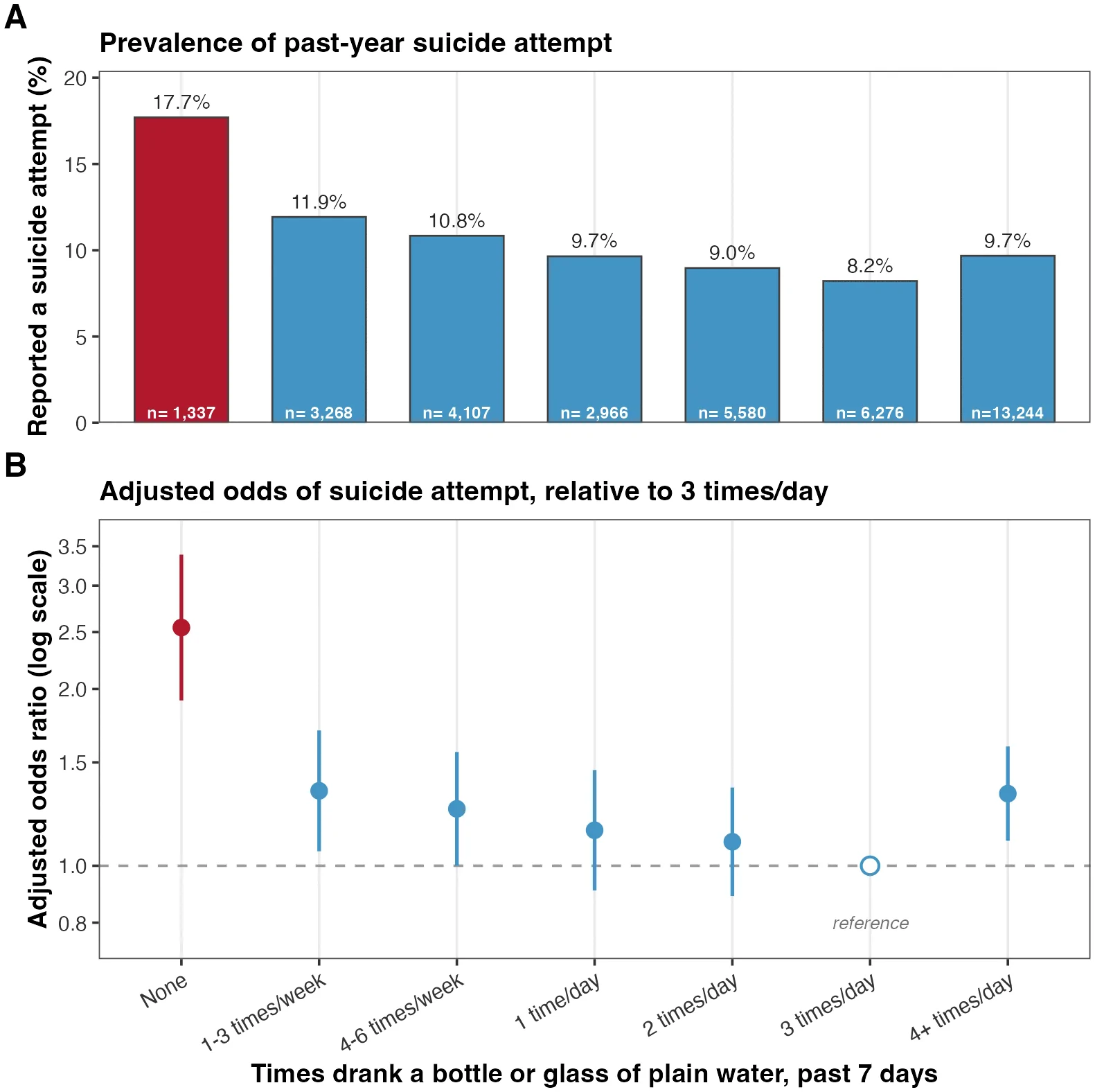
Association between plain-water consumption and past-year suicide attempt across the full ordinal item, pooled 2017-2021 (n = 36,779, averaged over 40 imputations). (A) Prevalence of past-year suicide attempt at each level of reported consumption. (B) Survey-weighted odds ratios adjusted for sex, race/ethnicity, grade, sexual orientation, Hispanic/Latino ethnicity and survey year, with 3 times/day as the reference category, this being the category with the lowest observed attempt prevalence. Bars and points for the zero category are shown in red.

Above the zero category there was no gradient. Among the 35,442 adolescents who reported drinking any plain water, the adjusted odds ratio per additional time per day is 1.01 (0.97–1.06, p = 0.60).

#### Adjustment against the risk domains.

Adjusted for demographic covariates alone the survey-weighted odds ratio is 2.10 (1.65–2.67). Adjusted additionally for each single domain it ranges from 1.45 (1.10–1.92) for trauma and victimization to 2.01 (1.56–2.59) for physical activity and sleep, with every interval excluding 1. Adjusted simultaneously for all domains it falls to 1.34 (0.97–1.85, p = 0.075) (Table 2).

**Table 2:** Survey-weighted odds ratios for reporting no plain water, from a separate unpenalized logistic regression for each domain (2017-2021, 40 imputations).

Domain model	Weighted OR for water (95% CI)	p	FMI
Demographics only	2.10 (1.65-2.67)	<0.001	0.12
Physical activity and sleep	2.01 (1.55-2.59)	<0.001	0.10
Suicidal ideation and distress	1.92 (1.40-2.63)	<0.001	0.11
Dietary habits	1.77 (1.39-2.27)	<0.001	0.13
Risk behaviors	1.65 (1.29-2.10)	<0.001	0.12
Substance use	1.69 (1.32-2.17)	<0.001	0.12
Trauma and victimization	1.45 (1.10-1.92)	0.009	0.10
All domains combined	1.34 (0.97-1.85)	0.075	0.11

Each row is a separate model containing the water indicator, demographic covariates, and the predictors in that domain, pooled by Rubin’s rules and accounting for the YRBS complex survey design. Unweighted counterparts are in S5 Table in S1 File. The demographics-only row is the estimation baseline. FMI = fraction of missing information.

### LASSO selection depends on the penalty rule

LASSO retention of the water indicator depended on the penalty rule (Fig 2). Under the discrimination-optimal rule, which chooses the penalty with the best cross-validated discrimination and therefore applies less shrinking, the indicator was retained in all six domain models and when all domains competed together (Fig 2A).

**Fig 2:**
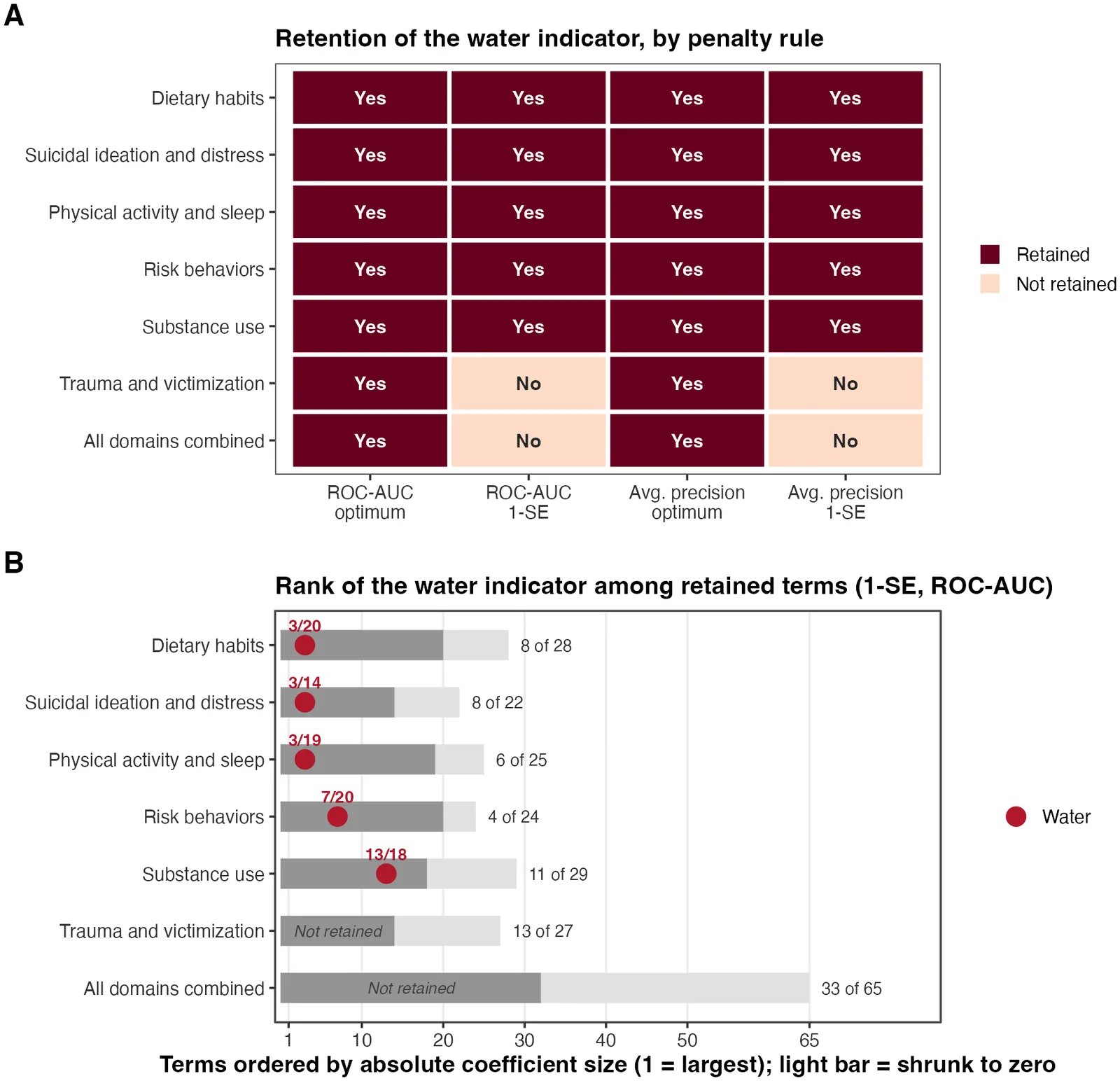
LASSO retention of the zero-water indicator (2017-2021). (A) Retention by tuning metric and penalty rule. Yes: retained in all 40 imputed training datasets (39 of 40 in the full model at the discrimination optimum). No: not retained. (B) One-standard-error fits tuned on ROC-AUC. Dark bar: terms retained, ordered by absolute coefficient. Light bar: terms shrunk to zero. Red point: water indicator, labeled as rank among retained terms. Right-hand counts are terms shrunk to zero of all terms in the model. Occasional non-zero coefficients in trauma and the full model are treated as imputation uncertainty (S7 Table in S1 File).

Under the one-standard-error rule, which takes a larger penalty and therefore keeps fewer predictors, the indicator was retained in five domain models: dietary habits, suicidal ideation and distress, physical activity and sleep, risk behaviors, and substance use. Among the terms retained in each of those models, ranked by the absolute size of the coefficient, it was third of 20 in the dietary model, third of 14 in the ideation-and-distress model, third of 19 in the physical-activity model, seventh of 20 in the risk-behavior model, and thirteenth of 18 in the substance-use model (Fig 2B). It was not retained in the trauma and victimization model, nor when all six domains competed together. Under that rule the full model retained a median of 32 of 65 terms. S6 Table in S1 File reports penalty and sparsity diagnostics for all 28 combinations. S7 Table in S1 File reports the rank of the water indicator among retained terms. S8 Table in S1 File reports the unpenalized odds ratio for the water indicator from logistic regression on the predictors retained under this rule, next to the corresponding shrunk LASSO odds ratio.

Because a LASSO model was fitted on each of the 40 imputed training datasets, we also checked whether this pattern held on every draw rather than on a single imputed sample. The five single-domain retentions under the one-standard-error rule appeared in all 40 imputations. In the trauma model and in the full model a non-zero coefficient appeared only in a minority of draws (at most 19 of 40 for trauma and 6 of 40 for the full model; 12 of 40 and 2 of 40 when tuned on average precision). We treat those occasional selections as a product of imputation uncertainty rather than as retention. These models were fitted without survey weights.

### Held-out discrimination and incremental value

Held-out discrimination ranged from 0.703 (dietary habits) to 0.923 (all domains combined) (Fig 3A). The models were well calibrated, with calibration slopes between 0.97 and 1.16 and intercepts within 0.24 of zero (S9 Table in S1 File).

**Fig 3:**
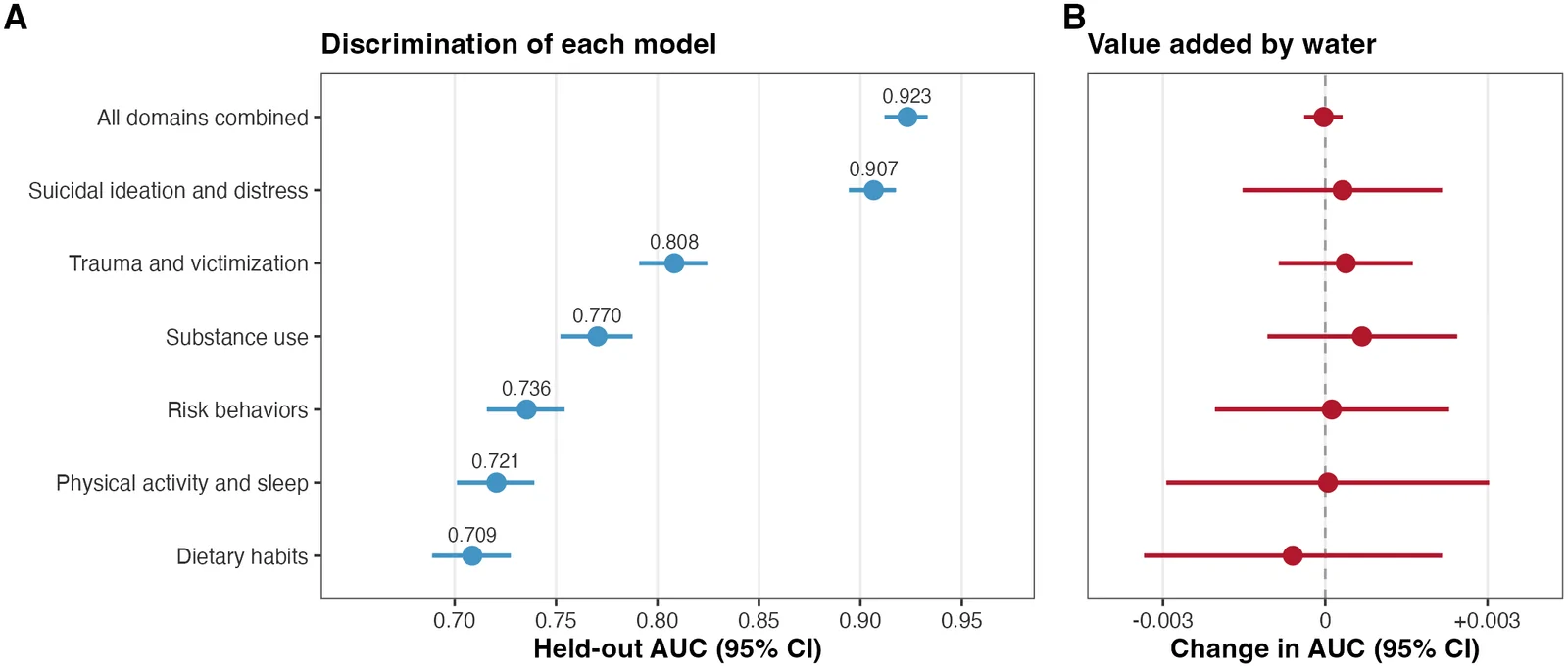
Held-out predictive performance (2017-2021; 9,195 held-out respondents; penalty at the discrimination optimum, tuned on ROC-AUC). (A) Area under the ROC curve for each model, with 95% confidence intervals. (B) Paired change in held-out AUC when the zero-water indicator is added to the same model on identical data, pooled across 40 imputations. Note the x-axis scale in panel B.

Fitting every model twice on identical data, with and without the water indicator, showed no incremental predictive value (Fig 3B). The largest change in held-out AUC observed across both tuning metrics and both selection rules, 28 model specifications in total, is 0.0011, the largest change in average precision is 0.0012, every confidence interval covers zero, and the smallest p-value is 0.40 (S10 Table in S1 File).

## Discussion

This study examined whether adolescents’ self-reported plain-water consumption is associated with suicide attempts when placed in direct competition with established risk factors across multiple theoretically grounded domains. Reporting no plain water in the past seven days was associated with past-year suicide attempt in each of the three survey cycles examined, and the water indicator was retained by penalized regression in five of the six risk domains considered separately, including the domain comprising suicidal ideation, planning, hopelessness and difficulty concentrating. The association did not persist under simultaneous adjustment for all domains, and it was confined to adolescents who reported drinking no plain water rather than graded across the consumption distribution. It also did not improve the discrimination of the penalized models in held-out data, that is, their ability to assign higher predicted risk to adolescents who reported a suicide attempt than to those who did not.

These findings contribute to emerging work on the role of environmental and behavioral exposures in adolescent suicide risk [14]. This perspective expands traditional models, which have focused primarily on individual psychopathology or have combined adolescent and adult samples despite the distinct developmental features that heighten suicide vulnerability in adolescence [1]. Prior ecological studies have linked water-related factors, such as naturally occurring lithium in public water supplies, to lower suicide rates in largely adult populations [21,22], but they measure the chemistry of water systems at the area level rather than individual drinking behavior and cannot speak to the behavior examined here. At the individual level, our results agree in direction and approximate magnitude with a survey of Korean adolescents in which the lowest water-intake group had elevated odds of suicide attempt [18]. By examining an adolescent sample and by placing the indicator in competition with established predictors, the present study extends this literature to an everyday behavior that has received little attention in suicide research.

Retention under penalization depended on the penalty rule. Under the discrimination-optimal rule the water indicator was retained in all six domain models and in the full model. Under the stricter one-standard-error rule it was retained in the dietary, ideation-and-distress, physical-activity, risk-behavior, and substance-use models, and was not retained in the trauma and victimization model or when all six domains competed together. Occasional non-zero coefficients in a minority of imputed datasets in those last two specifications are treated as imputation uncertainty rather than as retention. This pattern parallels the unpenalized estimates, in which the indicator remained associated with suicide attempt after adjustment for each domain separately and attenuated toward the null under simultaneous adjustment. Retention alongside suicidal ideation, planning and hopelessness is notable, since those items are among the strongest known correlates of adolescent suicide attempt. That domain comprises four items, however, and cannot represent the full range of psychiatric and social conditions relevant to suicide risk, so retention alongside them does not establish independence from psychopathology more broadly.

The association was confined to the lowest category of the consumption item. Among adolescents who reported drinking any plain water, consumption carried no information about suicide attempt. This pattern is not consistent with a graded physiological mechanism, which would predict risk declining across the consumption distribution, and it differs from the Korean study, which reported an inverse dose-dependent pattern in which intermediate intake carried intermediate risk [18]. The two exposure measures are not identical, since that item counts glasses per day and the YRBS item counts occasions per day in coarser categories, and a gradient in the underlying quantity could be detected by one measure and not the other. The present results nonetheless indicate that reporting no plain water and reporting little plain water are better treated as distinct exposures than as adjacent points on a single scale, and that the full response distribution is worth reporting rather than a dichotomy specified in advance. Lower plain-water intake might also be downstream of physical inactivity, but interactions with physical activity and sports team participation were not statistically significant and the association was of similar magnitude within every stratum of both (S11 Table in S1 File).

The association carried no incremental predictive value. Adding the water indicator to any domain model left held-out discrimination unchanged (S10 Table in S1 File). This does not contradict the observed association, for two reasons. First, the exposure is rare, so even a substantial odds ratio moves too few adolescents across a risk threshold to shift a rank-based summary such as the area under the ROC curve, an insensitive instrument for low-prevalence markers [29,30]. Second, the indicator overlaps with predictors already in the models rather than carrying information separate from them, which is what the attenuation under simultaneous adjustment showed. A marker can therefore be robustly associated with an outcome and still be redundant for prediction.

Nor are the domain models themselves usable for screening at this outcome prevalence. Reaching 80% sensitivity with the models built from behavioral items alone requires flagging 39% to 57% of students, and fewer than one in five of those flagged reported an attempt (S12 Table in S1 File). The two specifications that perform better at these thresholds both contain suicidal ideation and planning, which lie close to the outcome itself.

Taken together, these results are consistent with the zero-water response functioning as a behavioral marker of circumstances already partly captured by established risk factors rather than as an independent contributor to risk. Several accounts remain compatible with the data and cannot be distinguished with the YRBS, including disengagement from routine self-care, constrained access to drinking water at home or at school, food insecurity, and substitution of other beverages for water. Distinguishing among them requires measures the survey does not carry, such as household resources, school water infrastructure, and a hydration measure not dependent on recall.

These results speak to the broader observation that limited progress in predicting adolescent suicidal behavior may partly reflect the narrow range of predictors conventionally examined [1,2], and they also qualify it. A novel correlate can be robustly associated with an outcome and still carry no incremental predictive value, and distinguishing the two requires the incremental-value analysis that studies proposing new markers frequently omit.

### Limitations

The temporal structure of the measures is the most serious limitation. The outcome refers to the past 12 months while the exposure refers to the past 7 days, so any attempt occurring more than seven days before the survey preceded the exposure window, and for those respondents the plain-water measure cannot be interpreted as antecedent. This design therefore does not support inferences about direction, and reverse causation, in which the aftermath of an attempt disrupts routine self-care, is as consistent with the data as the ordering that treats plain-water consumption as antecedent.

Missing data are a second limitation. Missingness on the plain-water item is 19.7%, it differs by outcome status, and 19.5% of respondents are excluded for a missing outcome. That exclusion is enriched for the exposure more than threefold, which does not bias the odds ratio on its own, but we cannot rule out that outcome missingness also depends on the outcome, which would. Multiple imputation assumes missingness is at random conditional on the imputation model, and these patterns must be argued rather than assumed. Retaining missingness as its own category leaves the estimate for the zero category essentially unchanged (S4 Table in S1 File), which bounds the problem but does not rule out informative missingness on the exposure. Missingness also rises from 12.0% in 2017 to 29.1% in 2021, while the odds ratio falls from 2.77 to 1.69, so the two trends cannot be separated with these data and the decline should not be read as evidence of a weakening association. Third, the exposure is a single self-reported item subject to recall error and to response-style effects in a long questionnaire. We have no information on water source or quality, no geographic identifiers, and no objective hydration measure. Fourth, the penalized selection models are unweighted, so selection results describe this sample rather than the U.S. adolescent population. The effect estimates we report are design-based, and the unweighted versions of the same models agree with them closely enough that no conclusion turns on the difference (S5 Table in S1 File). Fifth, the analysis covers 2017–2021, the cycles carrying the full measure set, so the findings describe that period and should not be assumed to hold in later cycles. Adolescent suicide risk and reported plain-water consumption have both shifted over time, and whether the association observed here persists is an empirical question that requires cycles administering the same predictors. Finally, LASSO retention identifies predictors that contribute in the presence of others under a particular penalty. Retention can reverse between two standard penalty rules, and it establishes neither substantive importance nor causal relevance.

## Conclusions

Among U.S. adolescents, reporting no plain-water consumption in the past seven days is associated with roughly twice the odds of a past-year suicide attempt. The association is present in all three national survey cycles, survives adjustment for each of six domains of established risk factors considered separately, and under a LASSO stress test is retained even in models that include variables previously associated with suicide. It is, however, confined eto the zero category and does not persist under simultaneous adjustment for all domains. These findings are exploratory and cross-sectional. They identify the zero-water response as a correlate worth understanding rather than a risk factor to be acted upon, and they do not support its use for screening or risk stratification.

## Supporting information

S1 File Supporting tables and figures. Compiled supporting information containing S1 and S2 Figs and S1–S12 Tables.
(DOCX)
