# Supplementary material for: Association between plain-water consumption and suicide attempts among U.S. adolescents: Evidence from the 2017–2021 Youth Risk Behavior Survey
Source: PLoS One. 2026 Oct 5;21(10):e0359065. doi: 10.1371/journal.pone.0359065 (PMC13637915; doi:10.1371/journal.pone.0359065)
Supplement: Supplementary file 1 [file pone.0359065.s001.docx]

**Supporting information: Association between plain-water consumption and suicide attempts among U.S. adolescents: Evidence from the 2017–2021 Youth Risk Behavior Survey**

These supporting tables and figures accompany the manuscript Association between plain-water consumption and suicide attempts among U.S. adolescents: Evidence from the 2017–2021 Youth Risk Behavior Survey Items are numbered as cited in the main text.

**# S1 Table. Missingness by variable**

Percentage of the 36,779 respondents in the analytic sample for whom each variable was not observed, before imputation. Variables observed for every respondent are omitted.

| **Variable** | **n missing** | **% missing** |
| --- | --- | --- |
| Texting Driving | 17,632 | 47.94 |
| Sexual Abuse By Partner | 17,552 | 47.72 |
| Driving Drinking | 17,448 | 47.44 |
| Physical Dating Violence | 14,687 | 39.93 |
| Difficulty Concentrating | 11,980 | 32.57 |
| No Sports Drinks | 10,637 | 28.92 |
| Ever Hallucinogenic Drugs | 10,551 | 28.69 |
| No Milk | 9,530 | 25.91 |
| More Than 10 Drinks | 8,803 | 23.93 |
| Sports Team | 8,484 | 23.07 |
| Ever Used Synthetic Marihuana | 8,248 | 22.43 |
| Ever Used Inhalant | 7,988 | 21.72 |
| Plain water, past 7 days | 7,236 | 19.67 |
| Ever Had Sex | 7,115 | 19.35 |
| Gun Carrying | 6,858 | 18.65 |
| Attended PEClass | 6,641 | 18.06 |
| Ever Used Injected Illegal Drug | 6,594 | 17.93 |
| No Other Veggies | 6,456 | 17.55 |
| Very Overweight | 6,413 | 17.44 |
| No Carrots | 6,404 | 17.41 |
| No Potatoes | 6,377 | 17.34 |
| No Soda | 6,357 | 17.28 |
| No Salad | 6,212 | 16.89 |
| Sexual Violence | 5,505 | 14.97 |
| Suicide Injury Medical Attention | 5,431 | 14.77 |
| Ever Used Cocaine | 5,238 | 14.24 |
| Ever Used Ecstasy | 5,165 | 14.04 |
| Seat Bealt Use | 5,041 | 13.71 |
| No Fruit Juice | 4,845 | 13.17 |
| Weapon Carrying School | 4,771 | 12.97 |
| Eightor More Hours Sleep | 4,123 | 11.21 |
| Forced Sexual Intercourse | 3,550 | 9.65 |
| Concussion Sports | 3,550 | 9.65 |
| Sexually Active | 3,339 | 9.08 |
| Sex 4 Or More Partners | 3,272 | 8.9 |
| Sex Before 13 | 3,202 | 8.71 |
| No Breakfast | 3,144 | 8.55 |
| Currently Vaping | 2,916 | 7.93 |
| Currently Alcohol | 2,798 | 7.61 |
| Sex Orientation | 2,517 | 6.84 |
| Currently Smoking Cigar | 2,087 | 5.67 |
| Currently Smokeless Tobacco | 1,853 | 5.04 |
| Three Or More Hours TV | 1,820 | 4.95 |
| Currently Smoking Cigarette | 1,634 | 4.44 |
| Three Or More Hours Video Games | 1,478 | 4.02 |
| Ever Used Metha | 1,460 | 3.97 |
| Ever Used Heroin | 1,415 | 3.85 |
| School Physical Fight | 1,273 | 3.46 |
| No Fruit | 1,241 | 3.37 |
| Offered Drugs School | 1,107 | 3.01 |
| Drinking Driver | 1,048 | 2.85 |
| Physical Activity | 983 | 2.67 |
| Pain Medicine | 977 | 2.66 |
| Suicide Plan | 913 | 2.48 |
| Race | 852 | 2.32 |
| Currently Use Marihuana | 596 | 1.62 |
| Threatened In School | 591 | 1.61 |
| Hispanic Latino | 573 | 1.56 |
| Bullying | 560 | 1.52 |
| Sex | 408 | 1.11 |
| Hopelessness | 372 | 1.01 |
| Suicide Ideation | 326 | 0.89 |
| Grade | 309 | 0.84 |
| Cyber Bullying | 267 | 0.73 |
| Unsafe At School | 160 | 0.44 |
| The binary no-water indicator and the other derived water variables are deterministic functions of the ordinal item and share its missingness, so they are reported once. | | |

**# S2 Table. Missingness by suicide-attempt status**

Percentage missing within each outcome group, with a chi-square test of independence between missingness and attempt status. The p_adj column applies a Benjamini-Hochberg correction across all variables tested. Rows are ordered by uncorrected p-value.

The pattern to note is that differential missingness is not confined to the plain-water item: it appears across the substance, trauma and risk domains. This is why the manuscript treats the missing-at-random assumption as something to be argued rather than assumed, and why a sensitivity analysis retaining missingness as its own exposure category is reported alongside the imputed estimates.

| Variable | % missing, no attempt | % missing, attempt | p | p (adjusted) |
| --- | --- | --- | --- | --- |
| Currently Smoking Cigarette | 3.76 | 10.66 | <0.001 | <0.001 |
| More Than 10 Drinks | 22.55 | 36.48 | <0.001 | <0.001 |
| Physical Dating Violence | 41.41 | 26.52 | <0.001 | <0.001 |
| Sexual Violence | 14.00 | 23.71 | <0.001 | <0.001 |
| Forced Sexual Intercourse | 8.88 | 16.63 | <0.001 | <0.001 |
| Suicide Injury Medical Attention | 13.87 | 22.89 | <0.001 | <0.001 |
| Sex 4 Or More Partners | 8.22 | 15.04 | <0.001 | <0.001 |
| Sex Before 13 | 8.04 | 14.71 | <0.001 | <0.001 |
| Currently Vaping | 7.30 | 13.59 | <0.001 | <0.001 |
| Sexually Active | 8.42 | 15.07 | <0.001 | <0.001 |
| Hopelessness | 0.79 | 3.04 | <0.001 | <0.001 |
| Currently Alcohol | 7.02 | 12.93 | <0.001 | <0.001 |
| Eightor More Hours Sleep | 10.51 | 17.53 | <0.001 | <0.001 |
| Seat Bealt Use | 13.02 | 19.93 | <0.001 | <0.001 |
| Sex | 0.91 | 2.93 | <0.001 | <0.001 |
| Bullying | 1.30 | 3.55 | <0.001 | <0.001 |
| Currently Use Marihuana | 1.39 | 3.66 | <0.001 | <0.001 |
| Cyber Bullying | 0.58 | 2.02 | <0.001 | <0.001 |
| Plain water, past 7 days | 19.02 | 25.57 | <0.001 | <0.001 |
| Concussion Sports | 9.17 | 14.03 | <0.001 | <0.001 |
| No Milk | 25.20 | 32.38 | <0.001 | <0.001 |
| No Breakfast | 8.10 | 12.61 | <0.001 | <0.001 |
| Sexual Abuse By Partner | 48.51 | 40.55 | <0.001 | <0.001 |
| Ever Had Sex | 18.76 | 24.67 | <0.001 | <0.001 |
| Suicide Ideation | 0.75 | 2.11 | <0.001 | <0.001 |
| School Physical Fight | 3.20 | 5.80 | <0.001 | <0.001 |
| Difficulty Concentrating | 31.93 | 38.36 | <0.001 | <0.001 |
| Unsafe At School | 0.34 | 1.26 | <0.001 | <0.001 |
| Offered Drugs School | 2.79 | 5.00 | <0.001 | <0.001 |
| Suicide Plan | 2.29 | 4.24 | <0.001 | <0.001 |
| Driving Drinking | 46.88 | 52.47 | <0.001 | <0.001 |
| Grade | 0.75 | 1.70 | <0.001 | <0.001 |
| Sports Team | 22.66 | 26.77 | <0.001 | <0.001 |
| Race | 2.18 | 3.58 | <0.001 | <0.001 |
| Ever Used Synthetic Marihuana | 22.05 | 25.81 | <0.001 | <0.001 |
| Ever Used Inhalant | 21.37 | 24.88 | <0.001 | <0.001 |
| No Sports Drinks | 28.54 | 32.35 | <0.001 | <0.001 |
| Sex Orientation | 6.63 | 8.75 | <0.001 | <0.001 |
| Texting Driving | 47.53 | 51.63 | <0.001 | <0.001 |
| Three Or More Hours Video Games | 3.86 | 5.41 | <0.001 | <0.001 |
| Hispanic Latino | 1.46 | 2.43 | <0.001 | <0.001 |
| Ever Hallucinogenic Drugs | 28.35 | 31.72 | <0.001 | <0.001 |
| Physical Activity | 2.56 | 3.72 | <0.001 | <0.001 |
| Three Or More Hours TV | 4.81 | 6.23 | <0.001 | <0.001 |
| Ever Used Ecstasy | 14.27 | 12.03 | <0.001 | <0.001 |
| Ever Used Cocaine | 14.46 | 12.25 | <0.001 | <0.001 |
| No Fruit | 3.28 | 4.24 | 0.003 | 0.004 |
| No Fruit Juice | 13.34 | 11.65 | 0.004 | 0.006 |
| No Soda | 17.10 | 18.92 | 0.006 | 0.008 |
| Pain Medicine | 2.59 | 3.23 | 0.027 | 0.035 |
| Threatened In School | 1.56 | 2.05 | 0.029 | 0.036 |
| Currently Smokeless Tobacco | 4.95 | 5.80 | 0.030 | 0.037 |
| Ever Used Metha | 3.90 | 4.62 | 0.037 | 0.045 |
| No Salad | 17.02 | 15.75 | 0.056 | 0.066 |
| No Carrots | 17.54 | 16.30 | 0.064 | 0.075 |
| Attended PEClass | 17.93 | 19.17 | 0.069 | 0.079 |
| No Other Veggies | 17.67 | 16.46 | 0.071 | 0.080 |
| Weapon Carrying School | 13.07 | 12.09 | 0.098 | 0.109 |
| Drinking Driver | 2.80 | 3.28 | 0.109 | 0.119 |
| No Potatoes | 17.44 | 16.46 | 0.146 | 0.157 |
| Very Overweight | 17.53 | 16.57 | 0.152 | 0.162 |
| Ever Used Injected Illegal Drug | 17.84 | 18.73 | 0.190 | 0.198 |
| Ever Used Heroin | 3.81 | 4.18 | 0.285 | 0.293 |
| Gun Carrying | 18.59 | 19.17 | 0.405 | 0.411 |
| Currently Smoking Cigar | 5.64 | 5.96 | 0.452 | 0.452 |

**# S1 Fig. Convergence of the imputation chains**

Trace of the chain mean and chain variance across the 20 iterations, for the plain-water item and the five imputed variables with the highest missingness. Each line is one chain. Convergence is a property of the algorithm and the model specification rather than of the number of imputations, so these chains were run under the specification used for the analysis with a reduced number of imputations; the datasets analyzed in the manuscript come from the full run of 40.

Well-behaved chains intermingle without trend or separation across iterations.

**# S2 Fig and S3 Table. Observed against imputed values of the plain-water item**

Distribution of the seven-level plain-water item among respondents for whom it was observed and among those for whom it was imputed.

The imputed distribution is shifted toward the lower categories, and this is the expected consequence of an imputation model that includes the outcome and the variables predicting non-response: respondents missing the plain-water item resemble those reporting low or no consumption more than they resemble the sample as a whole. An imputation model that reproduced the observed distribution exactly would be the concerning result here, because it would indicate that the model had learned nothing about who is missing.

**S3 Table Observed against imputed distribution of the plain-water item**

| **Plain water, past 7 days** | **Observed (%)** | **Imputed (%)** |
| --- | --- | --- |
| None | 3.63 | 4.14 |
| 1-3 times/week | 8.64 | 9.69 |
| 4-6 times/week | 11.04 | 11.76 |
| 1 time/day | 7.96 | 8.38 |
| 2 times/day | 15.18 | 15.27 |
| 3 times/day | 17.36 | 16.96 |
| 4+ times/day | 36.19 | 33.81 |

**# S4 Table. Sensitivity analyses on the exposure**

Three alternative treatments of the plain-water item, each reported against the primary estimate.

All models adjust for sex, race/ethnicity, grade, sexual orientation, Hispanic/Latino ethnicity and survey year. In the final two rows the reference category is any reported plain water, and the missing category is estimated rather than imputed. The estimate for the zero category is essentially unchanged when missingness is modeled explicitly, which bounds the influence of the imputation without ruling out informative missingness.

| Analysis | OR (95% CI) |
| --- | --- |
| Primary: imputed exposure, survey-weighted | 2.10 (1.65-2.67) |
| Imputed exposure, unweighted | 1.90 (1.61-2.24) |
| Complete case on the plain-water item, survey-weighted | 2.26 (1.75-2.93) |
| Missingness retained as its own category: No water | 2.07 (1.71-2.50) |
| Missingness retained as its own category: Missing | 1.34 (1.22-1.47) |

**# S5 Table. Weighted against unweighted domain-adjusted odds ratios**

The unpenalized models of Table 2 in the manuscript, fitted with and without survey weights. The LASSO models are unweighted; this table is the comparison that bears on whether that choice moves the estimates.

| Domain model | Weighted OR for water (95% CI) | p, weighted | Unweighted OR for water (95% CI) | p, unweighted |
| --- | --- | --- | --- | --- |
| Demographics only | 2.10 (1.65-2.67) | <0.001 | 1.90 (1.61-2.24) | <0.001 |
| Physical activity and sleep | 2.01 (1.55-2.59) | <0.001 | 1.78 (1.50-2.10) | <0.001 |
| Suicidal ideation and distress | 1.92 (1.40-2.63) | <0.001 | 1.69 (1.36-2.09) | <0.001 |
| Dietary habits | 1.77 (1.39-2.27) | <0.001 | 1.63 (1.37-1.95) | <0.001 |
| Risk behaviors | 1.65 (1.29-2.10) | <0.001 | 1.50 (1.26-1.80) | <0.001 |
| Substance use | 1.69 (1.32-2.17) | <0.001 | 1.51 (1.26-1.81) | <0.001 |
| Trauma and victimization | 1.45 (1.10-1.92) | 0.009 | 1.32 (1.10-1.60) | 0.003 |
| All domains combined | 1.34 (0.97-1.85) | 0.075 | 1.18 (0.93-1.50) | 0.177 |

**# S6 Table. Penalty and sparsity diagnostics**

For each of the 28 model specifications, the median selected penalty across the 40 imputations, the number of fits in which the selected penalty landed on a boundary of the tuning grid, and the median number of non-zero terms.

Fits at the lower boundary are the ones that matter for interpretation: there the penalty is effectively zero, essentially no shrinkage occurs, and retention of any given predictor carries little information. This occurs only under the discrimination-optimal rule. Under the one-standard-error rule no fit reached either boundary in any of the 560 fits, which is why the manuscript rests its selection statements on that rule.

| **Model** | **Metric** | **Rule** | **Median penalty** | **At lower bound** | **At upper bound** | **Non-zero terms** | **CV metric** |
| --- | --- | --- | --- | --- | --- | --- | --- |
| Substance use | Average precision | 1-SE | 5.18e-03 | 0/40 | 0/40 | 18 of 29 | 0.3512 |
| Trauma and victimization |  |  | 7.54e-03 | 0/40 | 0/40 | 14 of 27 | 0.4110 |
| Dietary habits |  |  | 3.56e-03 | 0/40 | 0/40 | 20 of 28 | 0.2330 |
| Physical activity and sleep |  |  | 5.18e-03 | 0/40 | 0/40 | 15 of 25 | 0.2409 |
| Suicidal ideation and distress |  |  | 4.37e-03 | 0/40 | 0/40 | 12 of 22 | 0.5236 |
| Risk behaviors |  |  | 5.18e-03 | 0/40 | 0/40 | 14 of 24 | 0.2995 |
| All domains combined |  |  | 5.18e-03 | 0/40 | 0/40 | 28 of 65 | 0.6273 |
| Substance use |  | Optimum | 7.91e-04 | 1/40 | 0/40 | 26 of 29 | 0.3565 |
| Trauma and victimization |  |  | 9.71e-04 | 1/40 | 0/40 | 23 of 27 | 0.4169 |
| Dietary habits |  |  | 1.00e-08 | 21/40 | 0/40 | 25 of 28 | 0.2371 |
| Physical activity and sleep |  |  | 7.91e-04 | 5/40 | 0/40 | 21 of 25 | 0.2453 |
| Suicidal ideation and distress |  |  | 7.91e-04 | 2/40 | 0/40 | 18 of 22 | 0.5297 |
| Risk behaviors |  |  | 3.73e-04 | 11/40 | 0/40 | 22 of 24 | 0.3053 |
| All domains combined |  |  | 7.91e-04 | 0/40 | 0/40 | 52 of 65 | 0.6339 |
| Substance use | ROC-AUC | 1-SE | 5.18e-03 | 0/40 | 0/40 | 18 of 29 | 0.7683 |
| Trauma and victimization |  |  | 7.54e-03 | 0/40 | 0/40 | 14 of 27 | 0.8007 |
| Dietary habits |  |  | 3.56e-03 | 0/40 | 0/40 | 20 of 28 | 0.7115 |
| Physical activity and sleep |  |  | 3.56e-03 | 0/40 | 0/40 | 19 of 25 | 0.7147 |
| Suicidal ideation and distress |  |  | 3.56e-03 | 0/40 | 0/40 | 14 of 22 | 0.9000 |
| Risk behaviors |  |  | 3.56e-03 | 0/40 | 0/40 | 20 of 24 | 0.7316 |
| All domains combined |  |  | 3.56e-03 | 0/40 | 0/40 | 32 of 65 | 0.9169 |
| Substance use |  | Optimum | 1.00e-08 | 25/40 | 0/40 | 27 of 29 | 0.7714 |
| Trauma and victimization |  |  | 5.43e-04 | 4/40 | 0/40 | 24 of 27 | 0.8041 |
| Dietary habits |  |  | 5.43e-04 | 4/40 | 0/40 | 25 of 28 | 0.7151 |
| Physical activity and sleep |  |  | 3.73e-04 | 9/40 | 0/40 | 22 of 25 | 0.7181 |
| Suicidal ideation and distress |  |  | 1.00e-08 | 23/40 | 0/40 | 20 of 22 | 0.9026 |
| Risk behaviors |  |  | 2.56e-04 | 18/40 | 0/40 | 22 of 24 | 0.7351 |
| All domains combined |  |  | 1.76e-04 | 19/40 | 0/40 | 61 of 65 | 0.9194 |

Penalties were tuned on a log-spaced grid of 50 values from 1e-8 to 1. The CV metric column reports the cross-validated value of the tuning metric in the column headed Metric, so values are comparable within a metric only.

**# S7 Table. Rank and penalized odds ratio of the zero-water indicator**

Under the one-standard-error rule. Rank is the median position of the water indicator among all retained terms in the model, ordered by the absolute size of the penalized coefficient within each imputation. Demographics are included in every model. Penalized odds ratios are shrunk by construction and are not effect estimates; those are in Table 2 of the manuscript. Rank is shown only for models in which the indicator was retained in all 40 imputations.

| **Model** | **Metric** | **Imputations retained** | **Rank** | **Retained terms** | **Median penalized OR** |
| --- | --- | --- | --- | --- | --- |
| Dietary habits | Average precision | 40/40 | 3 | 20 | 1.50 |
| Suicidal ideation and distress |  | 40/40 | 3 | 12 | 1.40 |
| Physical activity and sleep |  | 40/40 | 3 | 15 | 1.55 |
| Risk behaviors |  | 40/40 | 7 | 14 | 1.32 |
| Substance use |  | 40/40 | 14.5 | 18 | 1.21 |
| Trauma and victimization |  | 12/40 | — | 16 | 1.04 |
| All domains combined |  | 2/40 | — | 34 | 1.09 |
| Dietary habits | ROC-AUC | 40/40 | 3 | 20 | 1.52 |
| Suicidal ideation and distress |  | 40/40 | 3 | 14 | 1.47 |
| Physical activity and sleep |  | 40/40 | 3 | 19 | 1.64 |
| Risk behaviors |  | 40/40 | 7 | 20 | 1.39 |
| Substance use |  | 40/40 | 13 | 18 | 1.23 |
| Trauma and victimization |  | 19/40 | — | 16 | 1.04 |
| All domains combined |  | 6/40 | — | 35 | 1.04 |

The median penalized odds ratio is computed over imputations in which the coefficient was non-zero. For trauma and the full model those values describe the occasional selections treated as imputation uncertainty in the manuscript, not retention

**# S8 Table. Shrunk and unpenalized odds ratios on the LASSO-selected set**

One-standard-error rule, tuned on ROC-AUC. For each imputed training dataset the original variables with at least one non-zero LASSO coefficient were entered in an unpenalized logistic regression of suicide attempt. The water odds ratio from those refits is pooled by Rubin’s rules. The LASSO odds ratio is the median across imputations and is shrunk by construction; it is not an effect estimate. Unpenalized estimates for trauma and the full model are omitted because the water indicator was not retained in those specifications.

| **Model** | **Imputations retained** | **Shrunk LASSO OR** | **Unpenalized OR (95% CI)** | **Selected variables** |
| --- | --- | --- | --- | --- |
| Dietary habits | 40/40 | 1.52 | 1.72 (1.41-2.10) | 12 |
| Suicidal ideation and distress | 40/40 | 1.47 | 1.78 (1.40-2.26) | 9 |
| Physical activity and sleep | 40/40 | 1.64 | 1.87 (1.55-2.26) | 12 |
| Risk behaviors | 40/40 | 1.39 | 1.60 (1.31-1.95) | 12 |
| Substance use | 40/40 | 1.23 | 1.55 (1.26-1.90) | 15 |
| Trauma and victimization | 19/40 | — | — | 13 |
| All domains combined | 6/40 | — | — | 28 |
|  |  |  |  |  |

Fits are unweighted and use the training partition, matching the LASSO models. Selected variables is the median number of original predictors in the unpenalized refit, including demographics. Among the imputations in which LASSO assigned water a non-zero coefficient, the pooled unpenalized odds ratio is 1.37 (1.11-1.70) in the trauma model (19 of 40) and 1.25 (0.94-1.65) in the full model (6 of 40); those selections are treated as imputation uncertainty in the manuscript.

**# S9 Table. Held-out performance, all specifications**

Discrimination, average precision, Brier score and calibration on the held-out partition (n = 9,195), for every specification fitted with and without the zero-water indicator.

| **Model** | **Metric** | **Rule** | **Water item** | **AUC (95% CI)** | **Avg. precision** | **Brier** | **Cal. slope** | **Cal. intercept** |
| --- | --- | --- | --- | --- | --- | --- | --- | --- |
| Substance use | Average precision | 1-SE | With | 0.764 (0.745-0.782) | 0.317 | 0.0757 | 1.12 | 0.17 |
| Substance use |  |  | Without | 0.763 (0.744-0.781) | 0.317 | 0.0758 | 1.13 | 0.18 |
| Trauma and victimization |  |  | With | 0.804 (0.786-0.820) | 0.394 | 0.0707 | 1.16 | 0.23 |
| Trauma and victimization |  |  | Without | 0.803 (0.786-0.820) | 0.394 | 0.0707 | 1.16 | 0.23 |
| Dietary habits |  |  | With | 0.703 (0.682-0.723) | 0.221 | 0.0809 | 1.07 | 0.07 |
| Dietary habits |  |  | Without | 0.704 (0.683-0.723) | 0.220 | 0.0809 | 1.07 | 0.07 |
| Physical activity and sleep |  |  | With | 0.711 (0.690-0.730) | 0.229 | 0.0806 | 1.14 | 0.21 |
| Physical activity and sleep |  |  | Without | 0.711 (0.690-0.730) | 0.228 | 0.0806 | 1.14 | 0.21 |
| Suicidal ideation and distress |  |  | With | 0.902 (0.889-0.914) | 0.555 | 0.0556 | 1.07 | 0.05 |
| Suicidal ideation and distress |  |  | Without | 0.901 (0.888-0.913) | 0.556 | 0.0556 | 1.07 | 0.04 |
| Risk behaviors |  |  | With | 0.730 (0.709-0.749) | 0.270 | 0.0784 | 1.11 | 0.14 |
| Risk behaviors |  |  | Without | 0.729 (0.709-0.749) | 0.270 | 0.0785 | 1.11 | 0.14 |
| All domains combined |  |  | With | 0.917 (0.904-0.929) | 0.623 | 0.0512 | 1.10 | 0.06 |
| All domains combined |  |  | Without | 0.918 (0.904-0.929) | 0.624 | 0.0511 | 1.09 | 0.06 |
| Substance use |  | Optimum | With | 0.770 (0.752-0.787) | 0.323 | 0.0754 | 0.99 | -0.08 |
| Substance use |  |  | Without | 0.769 (0.751-0.787) | 0.323 | 0.0754 | 0.99 | -0.09 |
| Trauma and victimization |  |  | With | 0.808 (0.791-0.825) | 0.397 | 0.0703 | 1.02 | -0.02 |
| Trauma and victimization |  |  | Without | 0.808 (0.790-0.824) | 0.398 | 0.0703 | 1.02 | -0.03 |
| Dietary habits |  |  | With | 0.709 (0.689-0.728) | 0.227 | 0.0806 | 0.97 | -0.13 |
| Dietary habits |  |  | Without | 0.709 (0.690-0.728) | 0.226 | 0.0806 | 0.98 | -0.11 |
| Physical activity and sleep |  |  | With | 0.720 (0.700-0.739) | 0.233 | 0.0802 | 1.02 | -0.04 |
| Physical activity and sleep |  |  | Without | 0.720 (0.700-0.739) | 0.233 | 0.0803 | 1.02 | -0.03 |
| Suicidal ideation and distress |  |  | With | 0.906 (0.893-0.917) | 0.558 | 0.0551 | 1.03 | 0.00 |
| Suicidal ideation and distress |  |  | Without | 0.906 (0.893-0.917) | 0.559 | 0.0551 | 1.03 | -0.01 |
| Risk behaviors |  |  | With | 0.735 (0.716-0.754) | 0.276 | 0.0781 | 0.97 | -0.12 |
| Risk behaviors |  |  | Without | 0.735 (0.716-0.754) | 0.276 | 0.0781 | 0.98 | -0.11 |
| All domains combined |  |  | With | 0.923 (0.911-0.933) | 0.635 | 0.0502 | 1.02 | -0.03 |
| All domains combined |  |  | Without | 0.923 (0.911-0.933) | 0.635 | 0.0502 | 1.02 | -0.03 |
| Substance use | ROC-AUC | 1-SE | With | 0.765 (0.746-0.783) | 0.318 | 0.0757 | 1.11 | 0.14 |
| Substance use |  |  | Without | 0.764 (0.745-0.782) | 0.317 | 0.0757 | 1.12 | 0.16 |
| Trauma and victimization |  |  | With | 0.804 (0.786-0.821) | 0.395 | 0.0706 | 1.14 | 0.19 |
| Trauma and victimization |  |  | Without | 0.804 (0.786-0.821) | 0.395 | 0.0706 | 1.14 | 0.20 |
| Dietary habits |  |  | With | 0.703 (0.682-0.723) | 0.222 | 0.0809 | 1.07 | 0.06 |
| Dietary habits |  |  | Without | 0.704 (0.683-0.724) | 0.220 | 0.0809 | 1.07 | 0.07 |
| Physical activity and sleep |  |  | With | 0.714 (0.693-0.733) | 0.231 | 0.0804 | 1.11 | 0.15 |
| Physical activity and sleep |  |  | Without | 0.714 (0.693-0.734) | 0.231 | 0.0805 | 1.11 | 0.15 |
| Suicidal ideation and distress |  |  | With | 0.903 (0.890-0.915) | 0.556 | 0.0554 | 1.06 | 0.03 |
| Suicidal ideation and distress |  |  | Without | 0.902 (0.889-0.914) | 0.557 | 0.0554 | 1.06 | 0.03 |
| Risk behaviors |  |  | With | 0.732 (0.712-0.751) | 0.273 | 0.0783 | 1.07 | 0.06 |
| Risk behaviors |  |  | Without | 0.731 (0.711-0.751) | 0.272 | 0.0783 | 1.08 | 0.08 |
| All domains combined |  |  | With | 0.919 (0.907-0.930) | 0.627 | 0.0509 | 1.08 | 0.04 |
| All domains combined |  |  | Without | 0.919 (0.907-0.930) | 0.627 | 0.0509 | 1.08 | 0.04 |
| Substance use |  | Optimum | With | 0.770 (0.752-0.788) | 0.323 | 0.0754 | 0.98 | -0.11 |
| Substance use |  |  | Without | 0.770 (0.751-0.787) | 0.323 | 0.0754 | 0.98 | -0.11 |
| Trauma and victimization |  |  | With | 0.808 (0.791-0.825) | 0.397 | 0.0703 | 1.02 | -0.04 |
| Trauma and victimization |  |  | Without | 0.808 (0.791-0.824) | 0.397 | 0.0703 | 1.02 | -0.04 |
| Dietary habits |  |  | With | 0.709 (0.689-0.728) | 0.227 | 0.0806 | 0.98 | -0.11 |
| Dietary habits |  |  | Without | 0.709 (0.690-0.728) | 0.226 | 0.0806 | 0.99 | -0.10 |
| Physical activity and sleep |  |  | With | 0.721 (0.701-0.739) | 0.233 | 0.0802 | 1.00 | -0.08 |
| Physical activity and sleep |  |  | Without | 0.721 (0.701-0.739) | 0.232 | 0.0803 | 1.01 | -0.06 |
| Suicidal ideation and distress |  |  | With | 0.907 (0.894-0.918) | 0.557 | 0.0551 | 1.03 | -0.01 |
| Suicidal ideation and distress |  |  | Without | 0.906 (0.894-0.917) | 0.559 | 0.0551 | 1.03 | -0.01 |
| Risk behaviors |  |  | With | 0.736 (0.716-0.754) | 0.276 | 0.0781 | 0.97 | -0.13 |
| Risk behaviors |  |  | Without | 0.735 (0.716-0.754) | 0.276 | 0.0781 | 0.98 | -0.12 |
| All domains combined |  |  | With | 0.923 (0.912-0.933) | 0.634 | 0.0503 | 1.01 | -0.05 |
| All domains combined |  |  | Without | 0.923 (0.912-0.933) | 0.634 | 0.0503 | 1.01 | -0.05 |

A calibration slope of 1 and intercept of 0 indicate perfect calibration. Downsampling was not used, so predicted probabilities are on the scale of the outcome prevalence.

**# S10 Table. Incremental value of the water indicator, all specifications**

Paired difference in held-out discrimination between the model containing the zero-water indicator and the same model without it, computed within each fit before pooling across imputations.

| **Model** | **Metric** | **Rule** | **AUC without** | **AUC with** | **Delta AUC (95% CI)** | **p** | **Delta avg. precision** | **Water dropped (%)** |
| --- | --- | --- | --- | --- | --- | --- | --- | --- |
| Substance use | Average precision | 1-SE | 0.7631 | 0.7641 | +0.0010 (-0.0026 to +0.0046) | 0.567 | +0.0008 | 0.0 |
| Trauma and victimization |  |  | 0.8035 | 0.8036 | +0.0000 (-0.0005 to +0.0006) | 0.906 | -0.0000 | 70.0 |
| Dietary habits |  |  | 0.7036 | 0.7026 | -0.0010 (-0.0062 to +0.0042) | 0.707 | +0.0008 | 0.0 |
| Physical activity and sleep |  |  | 0.7105 | 0.7106 | +0.0001 (-0.0053 to +0.0054) | 0.986 | +0.0011 | 0.0 |
| Suicidal ideation and distress |  |  | 0.9015 | 0.9020 | +0.0005 (-0.0020 to +0.0030) | 0.694 | -0.0011 | 0.0 |
| Risk behaviors |  |  | 0.7293 | 0.7298 | +0.0005 (-0.0024 to +0.0034) | 0.747 | +0.0002 | 0.0 |
| All domains combined |  |  | 0.9176 | 0.9174 | -0.0002 (-0.0017 to +0.0012) | 0.766 | -0.0006 | 87.5 |
| Substance use |  | Optimum | 0.7693 | 0.7700 | +0.0006 (-0.0014 to +0.0026) | 0.528 | -0.0001 | 0.0 |
| Trauma and victimization |  |  | 0.8079 | 0.8082 | +0.0003 (-0.0009 to +0.0015) | 0.619 | -0.0003 | 0.0 |
| Dietary habits |  |  | 0.7093 | 0.7088 | -0.0005 (-0.0034 to +0.0024) | 0.741 | +0.0009 | 0.0 |
| Physical activity and sleep |  |  | 0.7200 | 0.7200 | +0.0000 (-0.0035 to +0.0035) | 0.998 | +0.0001 | 0.0 |
| Suicidal ideation and distress |  |  | 0.9059 | 0.9058 | -0.0000 (-0.0022 to +0.0021) | 0.969 | -0.0011 | 0.0 |
| Risk behaviors |  |  | 0.7352 | 0.7354 | +0.0002 (-0.0022 to +0.0025) | 0.894 | -0.0003 | 0.0 |
| All domains combined |  |  | 0.9227 | 0.9227 | -0.0000 (-0.0007 to +0.0006) | 0.894 | +0.0000 | 2.5 |
| Substance use | ROC-AUC | 1-SE | 0.7637 | 0.7649 | +0.0011 (-0.0015 to +0.0039) | 0.397 | +0.0009 | 0.0 |
| Trauma and victimization |  |  | 0.8040 | 0.8042 | +0.0002 (-0.0010 to +0.0015) | 0.706 | +0.0002 | 52.5 |
| Dietary habits |  |  | 0.7035 | 0.7031 | -0.0005 (-0.0038 to +0.0029) | 0.784 | +0.0012 | 0.0 |
| Physical activity and sleep |  |  | 0.7140 | 0.7136 | -0.0004 (-0.0045 to +0.0037) | 0.857 | +0.0001 | 0.0 |
| Suicidal ideation and distress |  |  | 0.9025 | 0.9030 | +0.0005 (-0.0020 to +0.0030) | 0.704 | -0.0008 | 0.0 |
| Risk behaviors |  |  | 0.7311 | 0.7318 | +0.0007 (-0.0022 to +0.0036) | 0.646 | +0.0008 | 0.0 |
| All domains combined |  |  | 0.9190 | 0.9190 | -0.0000 (-0.0012 to +0.0011) | 0.988 | +0.0000 | 80.0 |
| Substance use |  | Optimum | 0.7697 | 0.7704 | +0.0007 (-0.0011 to +0.0024) | 0.444 | -0.0002 | 0.0 |
| Trauma and victimization |  |  | 0.8080 | 0.8083 | +0.0004 (-0.0009 to +0.0016) | 0.550 | -0.0006 | 0.0 |
| Dietary habits |  |  | 0.7092 | 0.7086 | -0.0006 (-0.0034 to +0.0022) | 0.670 | +0.0008 | 0.0 |
| Physical activity and sleep |  |  | 0.7205 | 0.7206 | +0.0001 (-0.0029 to +0.0030) | 0.974 | +0.0002 | 0.0 |
| Suicidal ideation and distress |  |  | 0.9063 | 0.9066 | +0.0003 (-0.0015 to +0.0022) | 0.737 | -0.0012 | 0.0 |
| Risk behaviors |  |  | 0.7353 | 0.7354 | +0.0001 (-0.0020 to +0.0023) | 0.911 | -0.0003 | 0.0 |
| All domains combined |  |  | 0.9232 | 0.9232 | -0.0000 (-0.0004 to +0.0003) | 0.860 | +0.0001 | 2.5 |
|  |  |  |  |  |  |  |  |  |

The final column reports the percentage of fits in which the penalty set the water coefficient to zero, so that the two models coincide and the paired difference is necessarily zero. Every interval in this table includes zero.

**# S11 Table. Association within strata of physical activity**

Reported in response to the possibility that lower plain-water intake is downstream of lower physical activity rather than a parallel marker of it. If that were the main pathway, the association should be concentrated among less active adolescents. Panel A is the association within each stratum; panel B is the interaction on the log-odds scale.

| A. Association within stratum |  |  |  |
| --- | --- | --- | --- |
| **Stratum** | **Adjusted OR (95% CI)** | **p** | **FMI** |
| Not physically active | 1.83 (1.52-2.20) | <0.001 | 0.14 |
| Physically active | 1.77 (1.19-2.64) | 0.005 | 0.29 |
| Not on a sports team | 1.78 (1.44-2.21) | <0.001 | 0.18 |
| On a sports team | 2.06 (1.54-2.77) | <0.001 | 0.28 |
|  |  |  |  |

Unweighted, pooled across 40 imputations by Rubin's rules. The association is of similar magnitude in every stratum.

| B. Interaction terms |  |  |
| --- | --- | --- |
| **Interaction** | **Log-odds (95% CI)** | **p** |
| No water × physically active | -0.06 (-0.50 to 0.38) | 0.788 |
| No water × on a sports team | 0.18 (-0.19 to 0.56) | 0.332 |
| Neither interaction is statistically significant. |  |  |

**# S12 Table. Operating characteristics at five thresholds**

Sensitivity, specificity, predictive values and the proportion of the held-out sample flagged, for every specification containing the water indicator, at five decision thresholds.

The threshold, not the tuning metric, is what governs the sensitivity-specificity trade-off. These rows are reported in full because a single favorable operating point would misrepresent what any of these models can do at this outcome prevalence: reaching 90% sensitivity in the strongest model requires flagging more than one adolescent in four, and the weaker single-domain models require flagging the majority of students.

| **Model** | **Operating point** | **Threshold** | **Sens** | **Spec** | **PPV** | **NPV** | **% flagged** |
| --- | --- | --- | --- | --- | --- | --- | --- |
| Substance use | 0.5 cutoff | 0.500 | 0.11 | 0.99 | 0.55 | 0.91 | 1.9 |
|  | 80% sensitivity | 0.069 | 0.80 | 0.60 | 0.17 | 0.97 | 44.0 |
|  | 90% sensitivity | 0.045 | 0.90 | 0.36 | 0.13 | 0.97 | 66.2 |
|  | Youden-optimal | 0.085 | 0.73 | 0.70 | 0.20 | 0.96 | 34.3 |
|  | Prevalence-matched | 0.095 | 0.69 | 0.73 | 0.21 | 0.96 | 30.6 |
| Trauma and victimization | 0.5 cutoff | 0.500 | 0.20 | 0.99 | 0.60 | 0.92 | 3.2 |
|  | 80% sensitivity | 0.066 | 0.80 | 0.66 | 0.20 | 0.97 | 38.7 |
|  | 90% sensitivity | 0.045 | 0.90 | 0.45 | 0.15 | 0.98 | 58.1 |
|  | Youden-optimal | 0.090 | 0.72 | 0.77 | 0.24 | 0.96 | 28.1 |
|  | Prevalence-matched | 0.095 | 0.70 | 0.78 | 0.25 | 0.96 | 26.2 |
| Dietary habits | 0.5 cutoff | 0.500 | 0.01 | 1.00 | 0.48 | 0.91 | 0.2 |
|  | 80% sensitivity | 0.065 | 0.80 | 0.46 | 0.13 | 0.96 | 56.8 |
|  | 90% sensitivity | 0.052 | 0.90 | 0.28 | 0.12 | 0.96 | 74.0 |
|  | Youden-optimal | 0.104 | 0.59 | 0.74 | 0.19 | 0.94 | 29.2 |
|  | Prevalence-matched | 0.095 | 0.61 | 0.71 | 0.18 | 0.94 | 32.3 |
| Physical activity and sleep | 0.5 cutoff | 0.500 | 0.01 | 1.00 | 0.44 | 0.91 | 0.3 |
|  | 80% sensitivity | 0.068 | 0.80 | 0.50 | 0.14 | 0.96 | 53.2 |
|  | 90% sensitivity | 0.051 | 0.90 | 0.29 | 0.12 | 0.96 | 73.1 |
|  | Youden-optimal | 0.093 | 0.66 | 0.69 | 0.18 | 0.95 | 34.5 |
|  | Prevalence-matched | 0.095 | 0.64 | 0.70 | 0.18 | 0.95 | 33.3 |
| Suicidal ideation and distress | 0.5 cutoff | 0.500 | 0.51 | 0.96 | 0.60 | 0.95 | 8.0 |
|  | 80% sensitivity | 0.154 | 0.80 | 0.90 | 0.44 | 0.98 | 17.1 |
|  | 90% sensitivity | 0.029 | 0.90 | 0.76 | 0.28 | 0.99 | 30.2 |
|  | Youden-optimal | 0.102 | 0.85 | 0.86 | 0.39 | 0.98 | 20.6 |
|  | Prevalence-matched | 0.095 | 0.85 | 0.86 | 0.38 | 0.98 | 21.1 |
| Risk behaviors | 0.5 cutoff | 0.500 | 0.06 | 0.99 | 0.58 | 0.91 | 1.0 |
|  | 80% sensitivity | 0.064 | 0.80 | 0.50 | 0.14 | 0.96 | 52.9 |
|  | 90% sensitivity | 0.047 | 0.90 | 0.29 | 0.12 | 0.97 | 72.8 |
|  | Youden-optimal | 0.095 | 0.66 | 0.72 | 0.20 | 0.95 | 31.6 |
|  | Prevalence-matched | 0.095 | 0.65 | 0.72 | 0.20 | 0.95 | 31.3 |
| All domains combined | 0.5 cutoff | 0.500 | 0.52 | 0.97 | 0.69 | 0.95 | 7.2 |
|  | 80% sensitivity | 0.194 | 0.80 | 0.92 | 0.50 | 0.98 | 15.3 |
|  | 90% sensitivity | 0.038 | 0.90 | 0.80 | 0.33 | 0.99 | 26.3 |
|  | Youden-optimal | 0.109 | 0.86 | 0.88 | 0.43 | 0.98 | 19.0 |
|  | Prevalence-matched | 0.095 | 0.87 | 0.87 | 0.41 | 0.98 | 20.0 |

Penalty tuned on ROC-AUC at the discrimination optimum. Values are averaged across the 40 imputations. The corresponding rows for the other three tuning and rule combinations are available in the analysis output.
